# Simplifying the protocol for low-pollution-risk, efficient mouse myoblast isolation and differentiation

**DOI:** 10.1007/s44307-025-00060-0

**Published:** 2025-03-11

**Authors:** Yi Luo, Jia-Dong Zhang, Xue-Gang Zhao, Wei-Cai Chen, Wan-Xin Chen, Ya-Rui Hou, Ya-Han Ren, Zhen-Dong Xiao, Qi Zhang, Li-Ting Diao, Shu-Juan Xie

**Affiliations:** 1 Biotherapy Center, The Third Affiliated Hospital of Sun Yat-Sen University, Guangzhou, 510630 China; 2 Department of Surgical Intensive Care, The Third Affiliated Hospital of Sun Yat-Sen University, Guangzhou, 510630 China; 3 Vaccine Research Institute of Sun Yat-Sen University, Third Affiliated Hospital of Sun Yat-Sen University, Guangzhou, 510630 China

**Keywords:** Primary myoblasts, Isolation, Differentiation

## Abstract

Myoblasts are the primary effector cells that play crucial roles in myogenesis and muscle regeneration following injury. However, isolating purified primary myoblasts from murine skeletal muscle poses challenges for junior researchers. Here, we present a simplified, low-risk, and optimized protocol for the extraction and enrichment of these myogenic progenitor cells. Additionally, we demonstrate that, compared to F10 (Ham’s F-10)-based medium, DMEM (Dulbecco’s Modified Eagle’s Medium)-based differentiation medium provides a more conducive environment for myoblasts differentiation. This enhancement improves the efficiency of myofiber formation and the expression of myogenic markers.

**Graphical Abstract:**

A convenient, low-pollution-risk, and expeditious methodology for isolating primary myoblasts has been developed. Comparative analysis of myoblast differentiation under DMEM and F10-based medium conditions revealed that myotubes formed in DMEM displayed a more robust and hypertrophic phenotype.

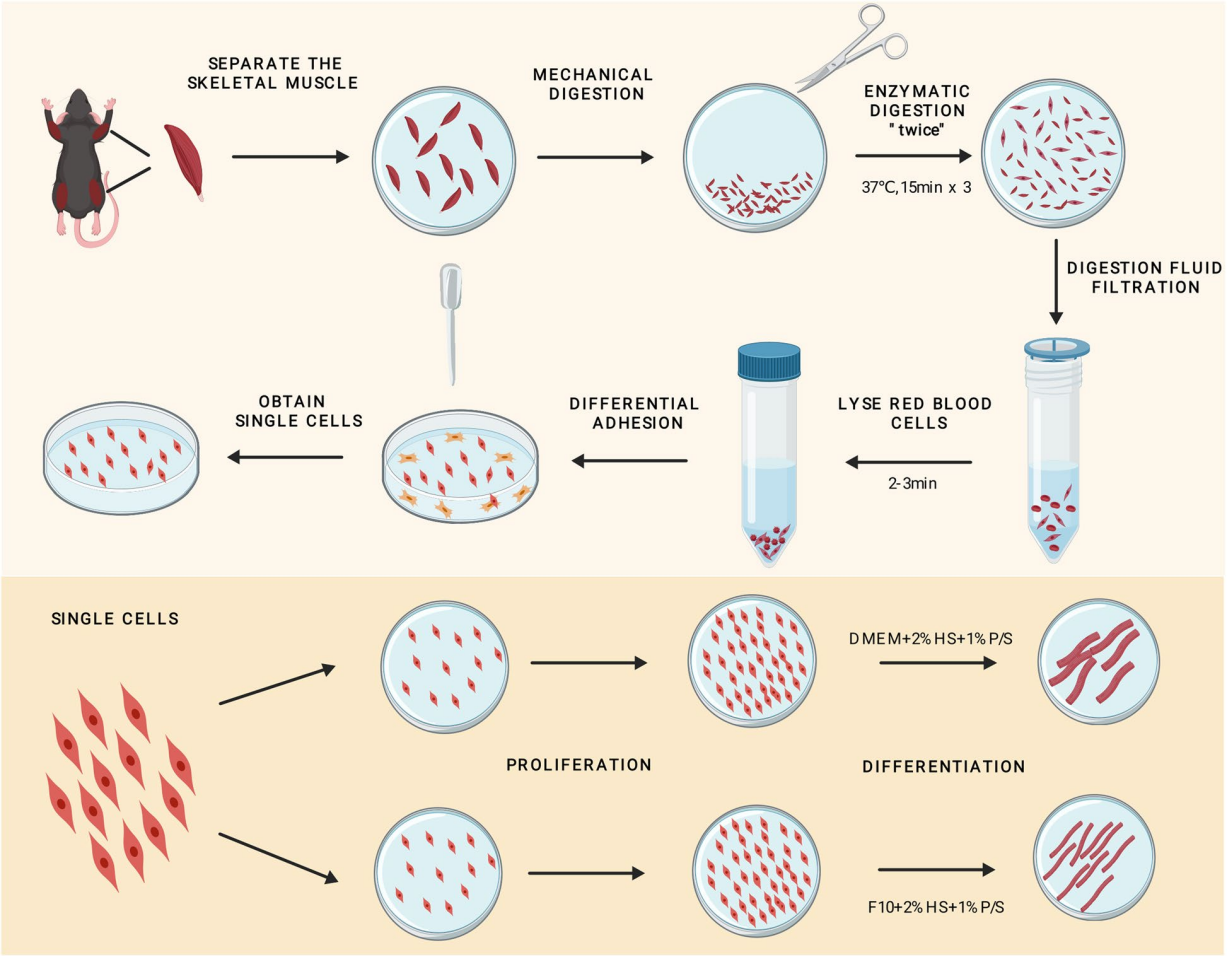

**Supplementary Information:**

The online version contains supplementary material available at 10.1007/s44307-025-00060-0.

## Introduction

Myoblasts, which originate from the proliferation of Muscle Stem Cells (MuSCs), play a crucial role in maintaining the physiological integrity of skeletal muscles. They possess the ability to proliferate, differentiate, fuse, and form muscle fibers, processes essential for muscle homeostasis and repair following injury. When muscle damage occurs, MuSCs are activated to proliferate. Some of these cells revert to a quiescent state to preserve the satellite cell pool, while others, termed myoblasts, participate in the regeneration of muscle tissues (Tidball 2017). Myoblasts can further proliferate, differentiate, and fuse to form nascent myotubes, which subsequently integrate with existing myotubes to expand the myonuclear domain, thereby enhancing cellular size and protein synthesis capacity (Feng et al., 2024).

Muscle differentiation is a multifaceted and complex process involving multiple stages and regulatory factors. The differentiation of myoblasts primarily consists of the following four distinct stages: (1) Activation stage: In this initial phase, quiescent muscle stem cells are stimulated to enter the cell cycle, giving rise to myoblasts (Kann et al. 2021). (2) Proliferation stage: Myoblasts undergo mitotic division to amplify their population. This process is regulated by a suite of molecules, including growth factors, cytokines, and extracellular matrix proteins. These molecules activate cell membrane receptors and trigger signaling pathways that promote cell proliferation (Nguyen et al. 2019). (3) Differentiation stage: Myoblasts gradually transition into mature muscle cells, beginning to express muscle-specific genes such as myosin heavy chain (MyHC) and members of the MEF2 protein family (Buckingham and Rigby 2014; Comai and Tajbakhsh 2014). (4) Fusion stage: Differentiated myoblasts fuse with existing muscle fibers, increasing the number of nuclei within the fibers and thereby enhancing the size and protein synthetic capacity of the entire cell (Tidball 2017). Myoblasts are essential for the processes of proliferation and differentiation following muscle injury.

The isolation of primary myoblasts is a multi-step process that is prone to contamination. In this study, we introduce an optimized protocol for extracting myoblasts from murine limb muscles, including their subsequent subculturing and differentiation. First, we optimized the experimental procedure by performing mouse dissections within a biosafety cabinet, significantly reducing the contamination rate during primary myoblast isolation. Next, we employed a differential adhesion method to separate contaminating cells, streamlining the extraction process. The entire experimental workflow is completed in approximately 6 h (h). Primary myoblasts obtained using this method exhibit high yield and viability. After 48 h of extraction and one passage, followed by an additional 48-h culture period, we harvested approximately 6.95 million myoblasts per 4–5-week-old mouse. Additionally, we compared the effects of two media-F10 and DMEM-based differentiation media (DM)- on myoblast differentiation. We found that DMEM-based DM promoted the formation of thicker myotubes and higher expression of differentiation markers, including MyHC and MEF2C.

## Material and methods

The following reagents and materials were purchased from their respective suppliers: PBS (ORIGENE, ZLI-9061), Horse serum (Gibco, 16050122), Fetal bovine serum (FBS, Gibco, 10099141C), Ham’s F-10 nutrient mix (F10, Gibco, 11550-043), Dulbecco’s modified Eagle’s medium (DMEM, Gibco, C11995500CP), Collagenase II (Gibco, 7101-015), Dispase (Gibco, 17105-041), Recombinant Human FGF-basic (hFGF, Abmole, M11108), Gelatin from porcine skin (sigma, 9000–70-8), Red Blood cell Lysis Buffer (Beyotime, C3702), Penicillin/Streptomycin (P/S, KeyGEN BioTECH, KGL2303-100), Amphotericin B (MCE, HY-B0221), Gentamicin (Sigma, E003632-1G), Anti-Myosin heavy chain (R&D systems, MAB4470), MEF2C mAb (Cell Signaling, 5030S), GAPDH mAb (Cell Signaling, 2118S), CD45 (PE/Cy7-CD45, eBioscience, 25–0451-82), CD31 (FITC-CD31, BioLegend, 102506), Scal (V450-Scal, BD, 560653), and VCAM1 (efluor660-VCAM1, eBioscience, 50–1061-80). Additionally, the following laboratory supplies were sourced from their respective manufacturers: 70-µm cell strainers, sterile (biolglx, 15–1070), 0.22 µm Filter (Millex, SLGPR33RB), 3 mL Transfer Pipet (biologix, 30-0138A1), Cell Culture Dish 100 mm × 20 mm Style (NEST, 704,001), Cell Culture Dish 150 mm × 25 mm Style (NEST, 715,001), Centrifuge Tube 50 mL, Economic Type, Clear, Sterile (NEST, 071824CH01). C57BL/6 J male mice, approximately 4 to 5 weeks old, were obtained from GemPharmatech Co., Ltd. (Jiangsu, China).The information of reagents and consumables can be found in Table 1.

### Preparation of muscle digestion media

Timing Approximately 10 min.

**Fig. 1:**
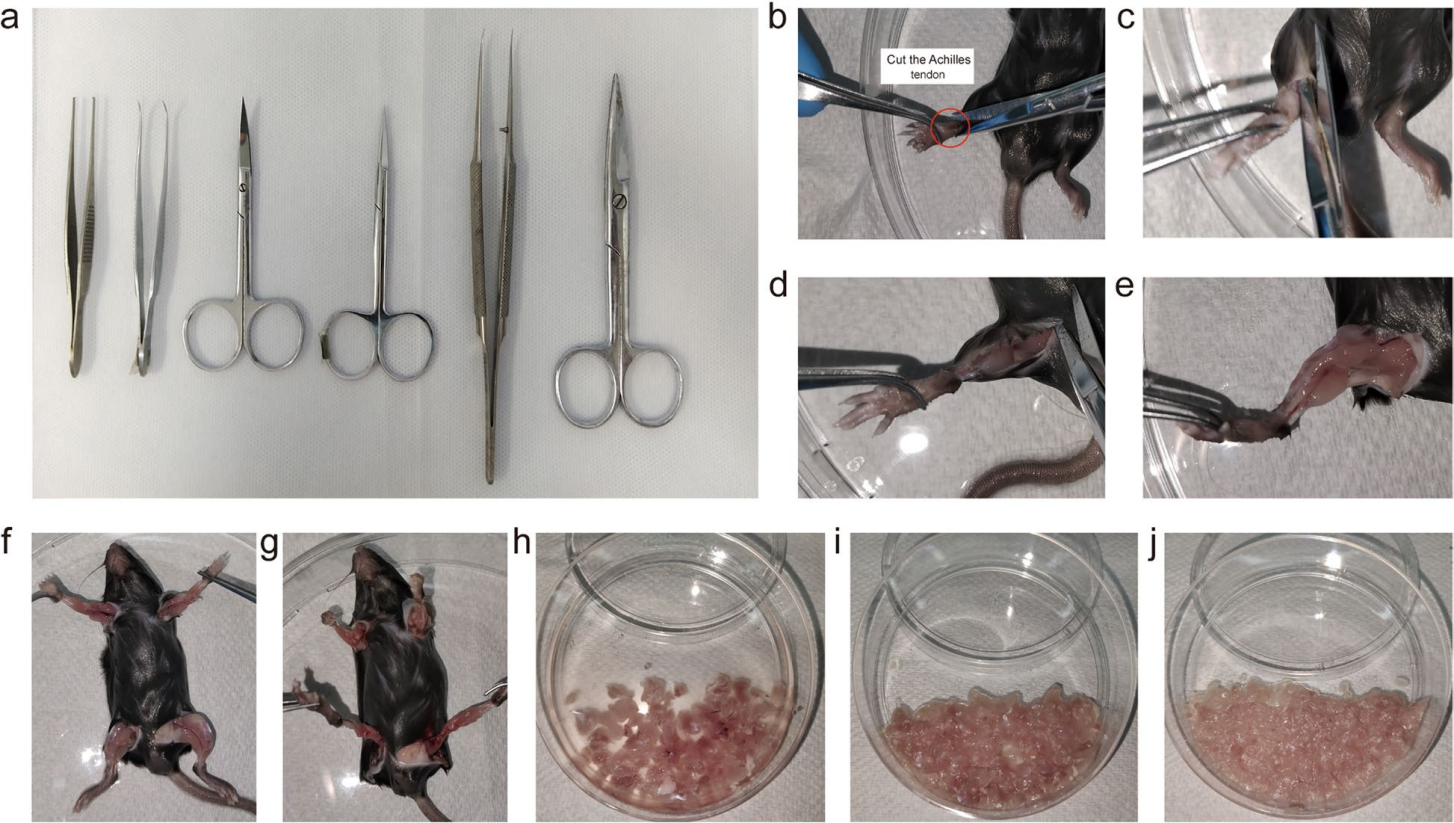
Isolation of skeletal muscle from the limbs of juvenile mice. **a** Instruments required for surgery. **b**-**g** Exposure and separation of skeletal muscle tissue. **h-j** Mechanical digestion of muscle tissue

1. In this study, a digestion solution containing 0.2% (w/v) collagenase type II and 0.2% (w/v) dispase was prepared using phosphate-buffered saline (PBS). The enzymatic digestion of skeletal muscle is a critical step in the extraction of primary myocytes. To ensure the efficiency of the digestion process, it is essential to pre-warm the digestion solution to 37 °C prior to use. The specific steps are as follows:
  - Accurately weigh the required amounts of collagenase type II and dispase powder into a 50 mL centrifuge tube;
  - Initially, add half of the final volume of PBS to dissolve the powders;
  - Filter the mixture through a 0.22 µm filter into a new tube and supplement with PBS to reach the final volume;
  - After disinfecting the exterior of the tube with alcohol, pre-warm the solution at 37°C for subsequent use.
2. In the biosafety cabinet, the following steps were conducted:
  - PBS was prepared containing 2% penicillin/streptomycin (P/S), 80μg/mL gentamicin, and 0.2% amphotericin B. Approximately 10 mL of this solution was aliquoted into a 10 cm cell culture dish for later use;
  - Surgical instruments (Fig. 1a) were introduced into the biosafety cabinet and exposed to UV light for 30 min with the lids open to sterilize them.

### Extraction of mouse skeletal muscle

Timing: Approximately 10–15 min per mouse.

This section describes the method for extracting skeletal muscle from mice, including the separation of muscle regions and methodologies.

3. After cleaning the work surface with 75% ethanol, prepare three clean beakers and fill them with 75% ethanol.
4. Following euthanasia of the mice by cervical dislocation, immerse the mouse in a beaker containing 75% ethanol for 5 mins. Subsequently, use forceps to remove the mouse and immerse it in another beaker, repeating the process for each beaker with a 5-min soak.
5. At the edge of the biosafety cabinet, transfer the mouse from the beaker to a 15 cm culture dish containing 75% ethanol. Then, within the cabinet, transfer it to a new 15 cm culture dish for dissection.
6. Cut the skin of the mouse at the ankle of the hind limb, insert scissors under the skin, and cut along the inner side of the hind limb towards the inguinal region. Use forceps to remove the skin, exposing the muscle.
7. Using new scissors and forceps, trim the skeletal muscle as much as possible, sequentially removing the muscles from all four limbs and placing them into a culture dish prepared with PBS containing antibiotics (Fig. 1b-g).
8. Change gloves, clean the dissection instruments with 75% ethanol, and repeat steps 6–7 for each additional mouse until all mice have been dissected.

#### CRITICAL:

1. When harvesting muscles, separate adipose tissue to avoid contamination with extraneous cells;
2. Harvest the hind limbs first, followed by the forelimbs. After processing one side, proceed to the other side while minimizing contact with hair.

### Muscle cleaning and mechanical digestion

Timing: Approximately 30 min per dish.

Mechanical digestion directly disrupts the extracellular matrix and cell–cell junctions, facilitating cell separation.

9. Rinse the muscle tissue three times with PBS supplemented with antibiotics to remove hair and blood.
10. After removing the PBS (to prevent dilution of collagenase), further mince the muscle with large surgical scissors until it reaches a paste-like consistency (continue mincing for approximately 20 mins) (Fig. 1h-j).

### Enzymatic digestion of skeletal muscle

Enzymatic digestion can more effectively break down cell-cell junctions and act more precisely on specific molecules, reducing non-specific damage to cells. However, it requires strict control over digestion time and conditions to avoid excessive digestion, which may lead to cell damage.

11. First Enzymatic Digestion
  - Add digestion buffer to the culture dish (for a 10 cm dish: 20 mL digestion buffer);
  - Spread the muscle evenly and digest in a 37°C incubator for 15 mins. During this period, expose the work surface of the biosafety cabinet to ultraviolet (UV) light;
  - Remove the dish and use a Pasteur pipette to triturate the muscle, ensuring uniform resuspension;
  - Repeat steps b and c twice, for a total digestion time of 45 mins (Figure 2a-c); (During the waiting period, prepare 10 cm culture dishes by coating them with 0.8% gelatin.)

**Fig. 2:**
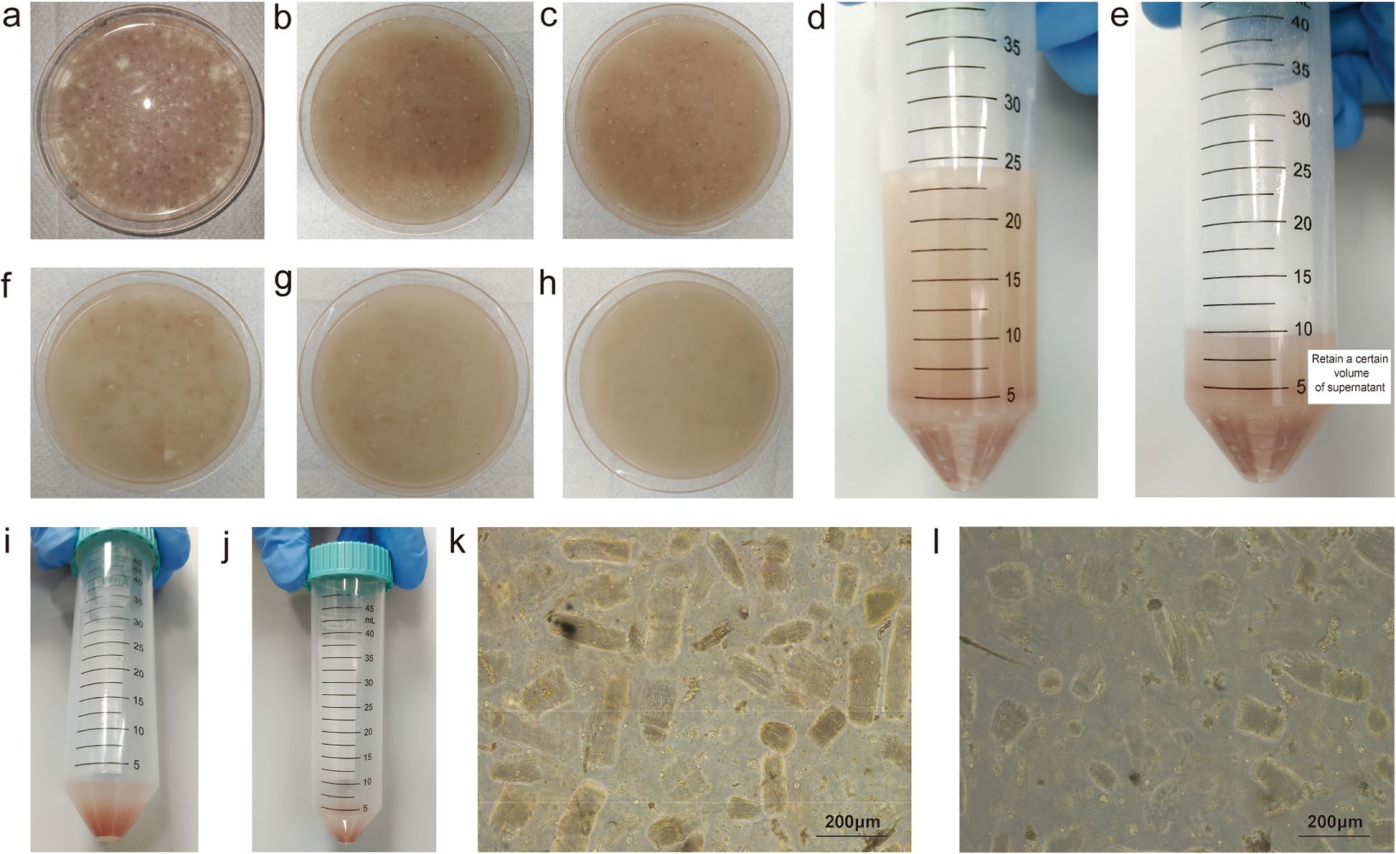
Isolation of single cells. **a**-**c** First enzymatic digestion. **d**-**e** Centrifugation after enzymatic digestion. **f**–**h** Second enzymatic digestion. **i** Cells before lysing red blood cells. **j** Cells after lysing red blood cells. **k** Cells before differential adhesion. **i** Cells after differential adhesion
  - Transfer the digested suspension to a 50 mL centrifuge tube and centrifuge at room temperature at 700g for 5 mins (Fig. 2d);
  - Discard the supernatant until 10 mL remains, and resuspend by gently pipetting the remaining digestion buffer (Fig. 2e).
12. Second Enzymatic Digestion
  - Pour the homogenized cell suspension into a new 10 cm culture dish, add 10 mL of digestion buffer, and mix evenly using a Pasteur pipette. Place the dish in a 37°C incubator for digestion for 15–20 mins. During this period, expose the work surface of the biosafety cabinet to UV light;
  - Remove the dish and use a Pasteur pipette to triturate the muscle, ensuring uniform resuspension;
  - Repeat step b twice, for a total digestion time of 45–60 mins (Fig. 2f-h).

### Lysis of red blood cells

The lysis of red blood cells (RBCs) is a critical step for enhancing the purity of isolated myocytes. RBCs can compete with myocytes for nutrients in the culture medium. Additionally, their presence may hinder the adhesion and proliferation of primary myocytes. Furthermore, the lysis of RBCs during culture can release hemoglobin and other cellular contents, which are detrimental to the culture environment and can compromise myocyte viability.

13. Add 10 mL of PBS to each dish and gently triturate the cells to dilute and homogenize the cell suspension. Filter the solution through a 70 µm cell strainer into a new 50 mL centrifuge tube to remove undigested tissue and cell clumps.
14. Centrifuge the cell suspension at 700 g for 5 min to pellet the cells. Discard the supernatant until 5 mL remains, and then resuspend the pellet by gently pipetting the remaining supernatant (Fig. 2i).
15. Add red blood cell lysis buffer at a 1:1 ratio to the resuspended cell pellet and incubate for 2–3 min to lyse the erythrocytes. After lysis, add PBS to a final volume of 50 mL to dilute the lysis buffer and neutralize its effects.
16. Centrifuge the treated cells at 700 g for 5 min to pellet the cells. Following centrifugation, a noticeable reduction in the number of erythrocytes should be observed due to lysis (Fig. 2j).
17. Remove the supernatant completely, and then resuspend the cell pellet by gently pipetting with 1 mL of pre-prepared complete culture medium (F10 supplemented with 20% FBS, 5% P/S, 80 μg/mL gentamicin, 0.2% amphotericin B, and 10 ng/mL hFGF). Transfer the resuspended cells into a new culture dish for further incubation and expansion.

### Differential adhesion for myocyte enrichment

After tissue digestion, a heterogeneous cell suspension containing various cell types is obtained. Differential adhesion is a technique that exploits the varying adhesive properties of different cell types to separate the cells of interest (myocytes) from non-target cells (such as fibroblasts and RBCs), thereby increasing the purity of the myocyte population (Fig. 2k-l).

18. Seed the mixed cell suspension into a non-coated dish and overlay it with 10 mL of culture medium containing 20% FBS. Incubate the dish at 37 °C with 5% CO2 for 40 min to allow for the preferential attachment of fibroblasts and other non-myocyte cells to the dish surface.
19. After the incubation period, carefully transfer the supernatant, which contains the non-adherent myocytes, to a new culture dish coated with extracellular matrix components. Culture the myocytes overnight at 37 °C with 5% CO2 to allow for further expansion.

#### Optional: flow cytometric profiling

- Cell Harvesting: Centrifuge the supernatant obtained after differential adhesion at 1,500 rpm for 3 mins;
- Suspension Preparation: Discard the supernatant, resuspend the pellet in 5 mL of PBS, and filter the suspension through a 70-μm cell strainer;
- Sample Partitioning: Aliquot the filtered suspension equally into six tubes. Centrifuge at 1,500 rpm for 3 mins, discard the supernatants, and resuspend the pellets in 100 μL of PBS supplemented with 0.3% BSA;
- Antibody Staining: Perform antibody incubation on ice using the following configurations:
  Unstained control: No antibodies.
  Single-color compensation controls: 0.5 μL of individual antibodies (CD45,CD31,Sca1,VCAM1) per tube.
  Experimental sample: 0.5 μL each of all four antibodies.
  Mix gently and incubate protected from light for 40 mins.
- Post-staining Processing: Add 1 mL of PBS to each tube, vortex gently, and centrifuge at 1,500 rpm for 3 mins. Resuspend the pellets in 300 μL of PBS for immediate acquisition on a flow cytometer.

The optimized isolation protocol yielded viable muscle stem cells (MuSCs, CD45^-^CD31^-^Sca1^-^VCAM1^+^) constituting 5.43% of the total cell population (Supplementary Figure 1), representing a 59.7% increase in purity compared to the 3.4% reported in prior study (Liu et al., 2015).

### Cell passage and differentiation

20. The cell status was assessed on the following day. If the cells exhibited good morphology and viability, the culturing was continued under standard conditions. If the cell status was unsatisfactory, indicating poor health or contamination, the cells were enzymatically digested and transferred to a new culture.
21. When cell confluence reached 70%, subculturing was performed: cells were digested with 0.25% trypsin for 2–3 min until they rounded up and detached from the culture dish. Digestion was then terminated by adding complete medium at twice the volume of trypsin used. The cell suspension was collected into a centrifuge tube and centrifuged at 1000 rpm for 3 min. The supernatant was discarded, and the cell pellet was resuspended in complete medium before being transferred to a new culture dish at a split ratio of 1:4 to 1:6. For Differentation, 3.5 × 105 cells/well were seed in six-well plate with culture medium containing 20% FBS.
22. Once the cell confluence exceeded 90%, the growth medium was replaced with a differentiation medium to initiate the differentiation process. The differentiation medium typically consisted of DMEM supplemented with 2% horse serum and 1% penicillin/streptomycin (P/S). Here, to compare the differences in cells cultured and differentiated in DMEM versus F10 medium, we compared the differentiation conditions as follows.

## Results and discussion

### Morphological changes of isolated primary myoblasts over time

**Fig. 3:**
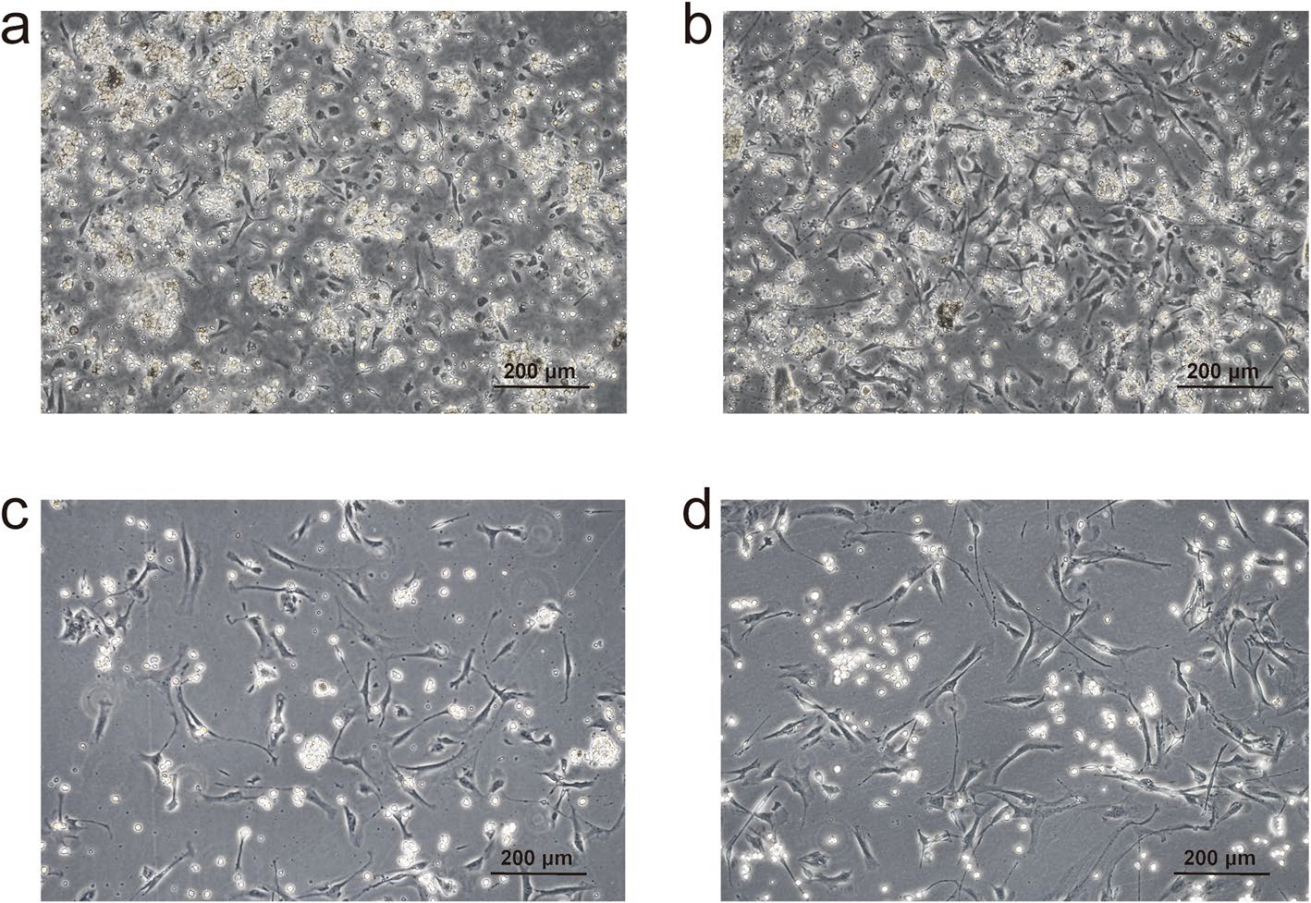
Morphology of primary cells at different time points. Images show the morphology of primary cells after isolation at: **a** 24 h, **b** 48 h, **c** 72 h (after one passage), and **d** 96 h

As illustrated in Fig. 3, within the first 24 h post-extraction, a considerable number of impurities were observed within the field of view. The isolated myoblasts exhibited a slightly spindle-shaped morphology at this stage (Fig. 3a). After culturing for an additional 24 h, the cells underwent morphological changes, becoming more elongated (Fig. 3b). At this point, corresponding to 48 h of culture, a passaging step was performed to enhance the purity of the myoblast population.

When the total culturing time reached 72 h, the number of contaminating cells had gradually diminished, and the majority of cells visible within the field of view were myoblasts, which now displayed a more uniform morphology (Fig. 3c). By the 96-h mark, the contaminating cells had been virtually eliminated, and the myoblasts were clearly discernible, exhibiting a characteristic elongated and bipolar shape within the field of view (Fig. 3d).

### Comparison of the differentiation efficacy of F10 and DMEM Media in Primary Myoblast Differentiation

**Fig. 4:**
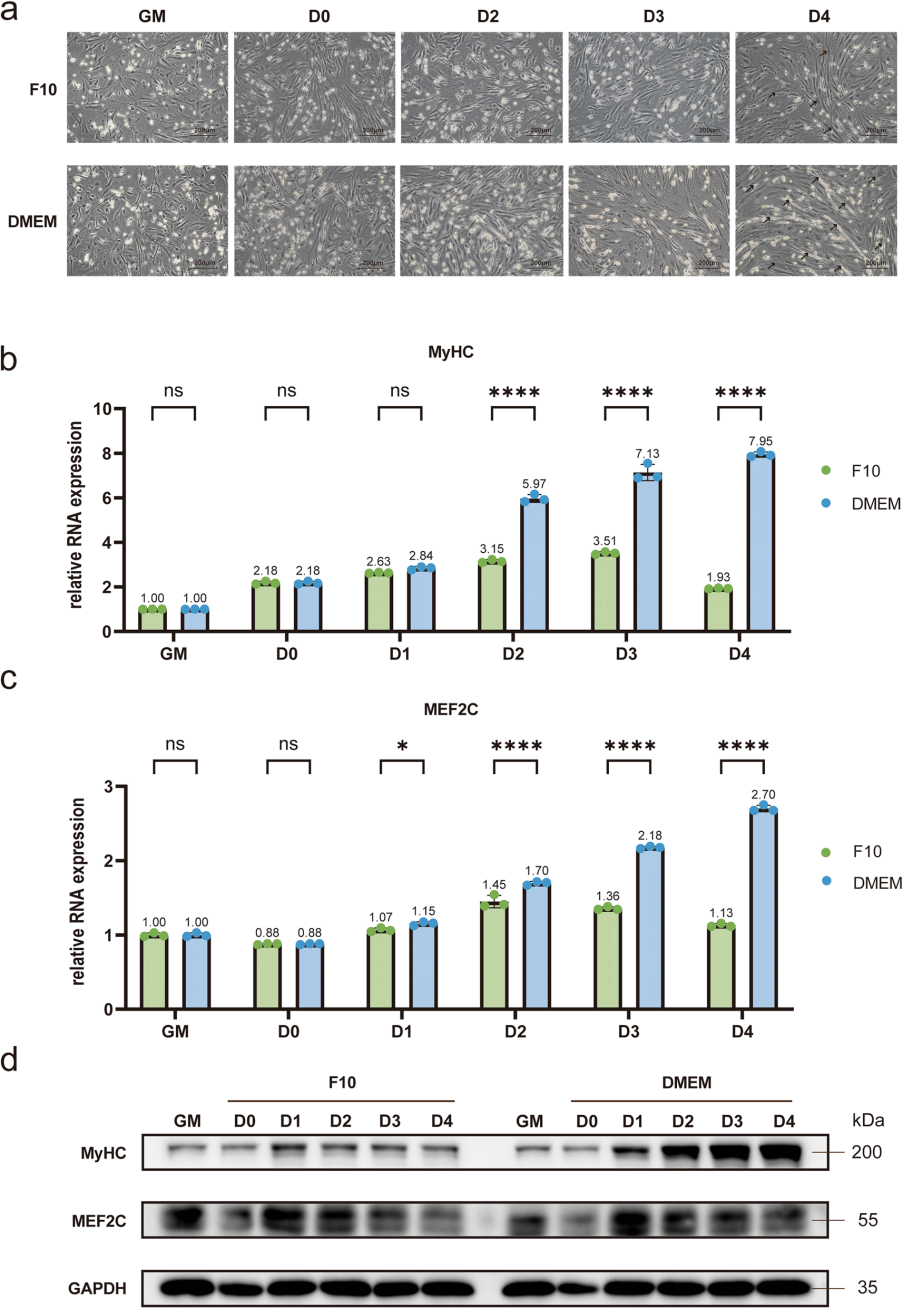
Differential phenotypes of myoblast differentiation under various media conditions. **a** Images showing differentiation at various time points using different differentiation media. **b**-**c** Quantification of RNA expression levels of differentiation markers in myoblasts cultured in different media at various time points. **b** The quantification of RNA expression levels of MyHC. **c** The quantification of RNA expression levels of MEF2C. Statistical significance was assessed using a two-tailed Student’s unpaired t-test. All bar graphs represent mean ± SD;* p* value: *p < 0.05, ****p < 0.0001. ns, no significant difference. **d** Western blot analysis of protein expression levels of differentiation markers in myoblasts cultured in different media at various time points. GAPDH gene was used as endogenous control

In the culturing of primary myoblasts, F10 medium is frequently employed during the proliferation stage, while DMEM is commonly used during the differentiation stage. F10 medium is recognized for its suitability in supporting the growth of primary myoblasts. However, it is unclear whether F10 medium can also support effective differentiation of these cells. Thus, we aimed to compare the differentiation efficacy of these two media when used as differentiation media (Fig. 4).

We seeded two groups of cells with an equal initial number of myoblasts. Using a six-well plate as an example, cells were seeded at a density of 3.5 × 10^5^ cells per well. Once the cell confluence exceeded 90%, the growth medium was replaced with either F10 or DMEM, each supplemented with 2% horse serum (HS), to initiate differentiation. Cell samples were collected during the growth period (GM) and from day 0 (D0) to day 4 (D4) of differentiation. We documented the morphological changes of the cells daily following the initiation of differentiation and assessed the fold changes in the protein and RNA expression levels of the differentiation markers MyHC and MEF2C.

Microscope observations revealed that when DMEM was used as the differentiation medium, the differentiated cells exhibited thicker myofilaments compared to those cultured in F10. On the fourth day of differentiation (D4), the cells cultured in DMEM displayed a relatively large number of myofilaments, with their positions marked by arrows in the figure (Fig. 4a). Quantitative PCR (qPCR) analysis indicated that the RNA expression levels of the differentiation markers MyHC and MEF2C were significantly upregulated under DMEM culturing conditions. On D4, compared to the growth period (GM), the RNA expression of MyHC in cells cultured with F10 showed a 1.93-fold increase, while MEF2C showed a 1.13-fold increase. In contrast, cells cultured with DMEM exhibited a 7.95-fold increase in MyHC and a 2.70-fold increase in MEF2C expression (Fig. 4b-c). The information of the primers used can be found in Table 2. Western blot (WB) results corroborated these findings, demonstrating a more pronounced upregulation of the protein levels of the myoblast differentiation markers when DMEM was used as the differentiation medium (Fig. 4d). Additionally, we performed grayscale analysis of the WB results using ImageJ software for quantitative assessment (Supplementary Fig. 2). These results demonstrate that DMEM medium is more effective as a differentiation medium for primary myoblasts, as it supports greater morphological differentiation and upregulation of key myogenic markers.

**Table 1:** KEY RESOURCES TABLE

**REAGENT or RESOURCE**	**SOURCE**	**IDENTIFIER**
**Chemicals, peptides, and recombinant proteins**		
PBS	ORIGENE	ZLI-9061
Hoses serum(HS)	gibco	16050122
Fetal bovine serum (FBS)	gibco	10099141C
Ham’s F-10 nutrient mix (F10)	gibco	11550-043
Dulbecco’s modified Eagle’s medium (DMEM), high glucose	gibco	C11995500CP
collagenase	gibco	17101-015
Trypsin-EDTA (0.25%)	gibco	25200-072
dispase	gibco	17105-041
Recombinant Human FGF-basic(hFGF)	Abmole	M11108
Gelatin from porcine skin	sigma	9000-70-8
Red Blood cell Lysis Buffer	Beyotime	C3702
Penicillin/Streptomycin(P/S)	Keygen BIO	KGL2303-100
Amphotericin B	MCE	HY-B0221
Gentamicin	Sigma	E003632-1G
Anti-Myosin hc	R&D ststems	MAB4470
MEF2C mAb	Cell Signaling	5030S
GAPDH mAb	Cell Signaling	2118S
PE/Cy7-CD45	eBioscience	25-0451-82
FITC-CD31	BioLegend	102506
V450-Scal	BD	560653
efluor660-VCAM1	eBioscience	50-1061-80
**Experimental models: Organisms**		
Mouse: C57BL/6J, male, approximately 5 weeks old	GemPharmatech	
**Other**		
70 µm cell strainers, sterile	biolglx	15-1070
0.22µm Filter	Millex	SLGPR33RB
3mL Transfer Pipet	biolglx	30-0138A1
Cell Culture Dish100mm×20mm Style	NEST	704001
Cell Culture Dish150mm×25mm Style	NEST	715001
Centrifuge Tube50mL,Economic Type,Clear,Sterile	NEST	071824CH01

**Table 2:** List of primers used in the study

**qPCR primer**	
	sequence (5'-->3')
MyHC-RT-qF	CGCAAGAATGTTCTCAGGCT
MyHC-RT-qR	GCCAGGTTGACATTGGATTG
Mef2c-RT-qF	ATCCCGATGCAGACGATTCAG
Mef2c-RT-qR	AACAGCACACAATCTTTGCCT
GAPDH-RT-qF	CGTCCCGTAGACAAAATGGT
GAPDH-RT-qR	TCAATGAAGGGGTCGTTGAT

### Troubleshooting

Problem 1: Cellular contamination during extraction.

Solutions:

1. Perform muscle tissue isolation within a biosafety cabinet to maintain a sterile environment.
2. Supplement the culture medium and PBS with 0.2% amphotericin to prevent fungal contamination.
3. Change gloves between handling different mice to prevent cross-contamination.

Problem 2: Large Tissue Fragments and slow filtration.

Solutions:

1. Replace the cell strainer if it appears clogged to improve the filtration rate.
2. Extend the manual dissociation time to ensure finer tissue fragments during the initial treatment.
3. Prolong the enzymatic digestion time to achieve a more complete breakdown of the tissue.
4. Pipette and mix the tissue fragments thoroughly during digestion to prevent aggregation and enhance digestion efficiency.

Problem 3: High heterogeneity of cells in the isolated solution.

Solution: Increase the number of differential adhesion steps to enrich for myoblasts and deplete fibroblasts and other non-muscle cells.

Problem 4: Low yield of myoblasts.

Solutions:

1. Increase the number of mouse samples to enhance the total yield of myoblasts.
2. Isolate muscles thoroughly to ensure complete tissue extraction and dissociation.

## Discussion

Myoblasts play an indispensable role in skeletal muscle growth, development, injury repair, and the maintenance of muscle homeostasis (Price et al. 2024; Liu et al. 2024). In the context of injury repair, myoblasts initially recognize damage and initiate the repair process. They then migrate toward the injury site along gradients of signaling molecules within the extracellular matrix. The repair is ultimately achieved through proliferation and differentiation (Sun et al. 2022). To further investigate the role of myoblasts in injury repair, it is imperative to develop a convenient method for myoblast isolation and to determine optimal differentiation conditions. The present study aims to establish a convenient and reliable approach for myoblast isolation and to identify the most suitable differentiation conditions by comparing different differentiation media.

This experimental protocol initially obtains single cells through mechanical digestion, enzymatic digestion, filtration, and differential adhesion methods. Compared with previous methods, this protocol offers several advantages. The differential adhesion approach is employed as an alternative to the conventional culture method for the acquisition of single cells (Vaughan et al., 2019). First, our protocol significantly reduces the risk of contamination. Compared with the method proposed by Vaughan M. et al., we conduct mouse dissection in a dedicated biosafety cabinet, minimizing the risk of sample exposure to external contaminants. Most importantly, the addition of 0.2% amphotericin B to the cell culture medium and PBS effectively decreases the contamination rate. These procedures have greatly enhanced the success rate of the experiment. Second, the process from isolating mouse muscle tissue to completing differential adhesion and obtaining single cells takes approximately 6 h, and the reagents and consumables used are relatively common. This study’s experimental process significantly shortens the time compared to the method of culturing and isolating single cells (Kim et al. 2020). Additionally, our protocol yields a greater number of cells with better viability. We utilized juvenile mice aged 4–5 weeks. After 48 h of extraction, followed by one passage and another 48 h of culture, approximately 6.95 million cells can be obtained from each mouse. However, in most studies conducted by other researchers, adult mice that are at least three months old were used. We quantified the proportion of muscle stem cells (MuSCs) using flow cytometry, which accounted for 5.43% of the total cell population (Supplementary Fig. 1). This represents a 59.7% increase in purity compared to the 3.4% reported in a previous study (Liu et al. 2015). MuSCs can be further sorted and purified for subsequent experiments if needed. Finally, we compared the effects of two types of media-F10- or DMEM-based differentiation media-on myoblast differentiation and found that DMEM as the differentiation medium resulted in thicker myotubes and higher expression levels of differentiation markers MyHC and MEF2C. On the fourth day after initiating differentiation (D4), compared with the growth medium (GM), the RNA and calculated protein expression levels of MyHC in the cells cultured in F10 were 1.93-fold and 1.10-fold, respectively, and those of MEF2C were 1.13-fold and 0.65-fold, respectively. In the cells cultured in DMEM, the fold changes of MyHC were 7.95 and 4.95, respectively, and those of MEF2C were 2.70 and 1.18, respectively.

After obtaining individual myoblasts, primary culture expansion is initially performed using F10 medium. Primary cells, directly isolated from tissues, retain many characteristics of their parent cells but are relatively delicate and have stringent culture environment requirements. F10 medium contains specific vitamins and growth factors. For instance, insulin modulates glucose uptake and utilization, while transferrin is involved in the transportation of iron ions, which are essential for numerous intracellular enzymatic systems.

The robustness of myotubes differentiated in DMEM medium likely stems from compositional differences between DMEM and F10 medium. DMEM medium contains a high concentration of glucose, providing substantial energy support required during myoblast differentiation (Xu et al. 2019). Additionally, DMEM medium is rich in amino acids, such as lysine and leucine, which are crucial for myosin heavy chain synthesis, a key component of myofilaments. Both DMEM and F10 media contain various vitamins, but there are differences in types and concentrations. For example, the vitamin B9 concentration is 4.0 mg/L in DMEM and only 1.3 mg/L in F10, with B9 playing a significant role in myoblast differentiation (Hwang et al. 2018). Finally, differences in inorganic salt content may also contribute to the observed differentiation outcomes. Calcium ions, for instance, are vital in cell signal transduction and regulate the myoblast differentiation process, including actin-myosin interactions and cytoskeletal reconfiguration (Minetti et al. 2011).

## Conclusions

Myoblasts are crucial in skeletal muscle physiology. The current study established a protocol for myoblast isolation that is time-efficient, has a low risk of contamination, and yields numerous viable cells. A comparison of F10 and DMEM media demonstrated that DMEM is superior for myoblast differentiation, resulting in thicker myotubes and higher expression of differentiation markers. Overall, this protocol is valuable for exploring the molecular and functional features of myoblasts, providing a reliable approach for related research.

## Supplementary Information

Supplementary Material 1. Supplementary Figure 1. Flow Cytometric Profiling (a) Profiles of the unstained activated MuSCs and their progeny. (b) Profiles of activated MuSCs and their progeny after antibody staining. The population hierarchy is depicted under the lots

Supplementary Material 2. Supplementary Figure 2. Quantification of protein expression levels of differentiation markers for myoblasts cultured in various differentiation media at different time points. (a) The quantification of protein expression levels of MyHC. (b) The quantification of protein expression levels of MEF2C. The statistical significance of differences was assessed using two-tailed Student’s unpaired t-test. All bar graphs are presented as the mean ± SD;* p* value: *p < 0.05, **p < 0.01, ***p< 0.001, ****p< 0.0001. ns, no significant difference.

## Data Availability

These resources are available upon reasonable request to the corresponding authors.
