# Supplementary figures and images for: Simplifying the protocol for low-pollution-risk, efficient mouse myoblast isolation and differentiation
Source: Adv Biotechnol (Singap). 2025 Mar 11;3(1):8. doi: 10.1007/s44307-025-00060-0 (PMC11896905; doi:10.1007/s44307-025-00060-0)

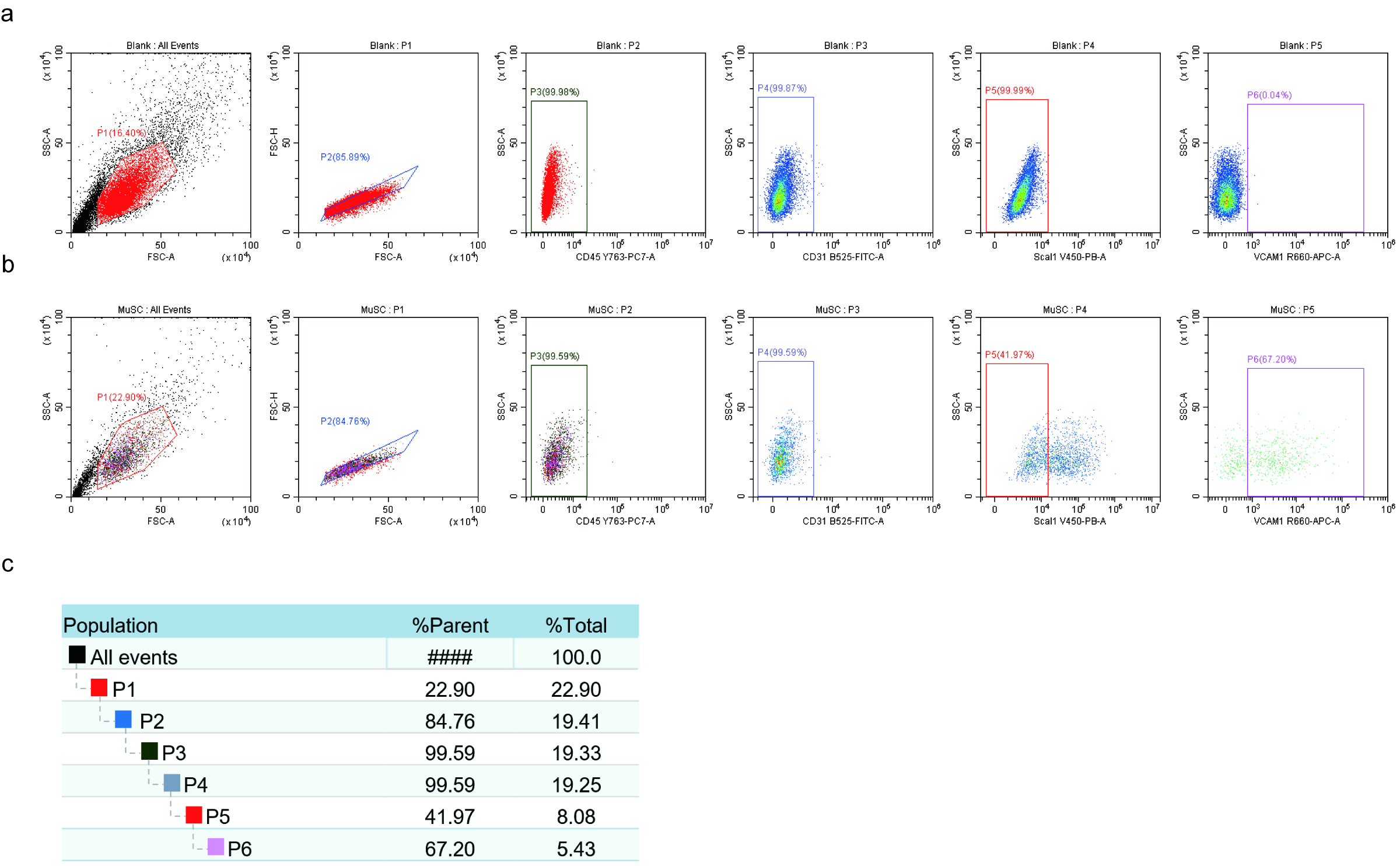

Supplement: Supplementary file 1 — Supplementary Material 1. Supplementary Figure 1. Flow Cytometric Profiling (a) Profiles of the unstained activated MuSCs and their progeny. (b) Profiles of activated MuSCs and their progeny after antibody staining. The population hierarchy is depicted under the lots [file 44307_2025_60_MOESM1_ESM.tif]

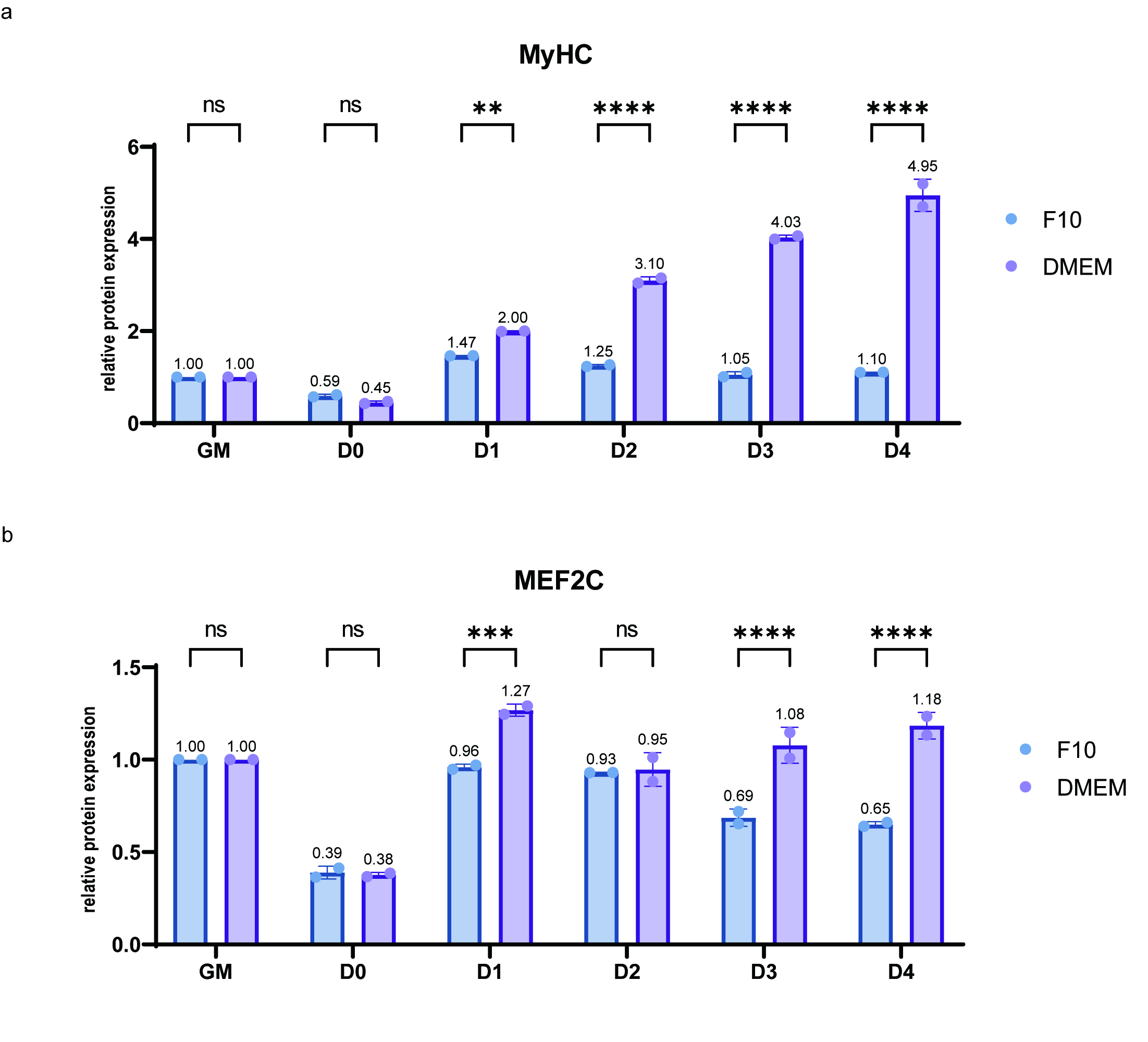

Supplement: Supplementary file 2 — Supplementary Material 2. Supplementary Figure 2. Quantification of protein expression levels of differentiation markers for myoblasts cultured in various differentiation media at different time points. (a) The quantification of protein expression levels of MyHC. (b) The quantification of protein expression levels of MEF2C. The statistical significance of differences was assessed using two-tailed Student’s unpaired t-test. All bar graphs are presented as the mean ± SD; p value: *p < 0.05, **p < 0.01, ***p< 0.001, ****p< 0.0001. ns, no significant difference. [file 44307_2025_60_MOESM2_ESM.tif]
